# Oncology Care Provision: Planning for Today, Tomorrow, and Years to Come

**DOI:** 10.3390/curroncol28060403

**Published:** 2021-11-17

**Authors:** Genevieve Chaput, Evan Lilly

**Affiliations:** 1 McGill University Health Centre, Division of Oncology and Family Medicine, McGill University, Montreal, QC H8S 3N5, Canada; 2 Vaudreuil-Soulanges Palliative Care Residence, Hudson, QC J0P 1H0, Canada; 3 Division of Oncology and Palliative Care, Bluewater Health, Sarnia, ON N7T 6S3, Canada; evanlilly11@gmail.com

Prior to the COVID-19 pandemic, the Canadian Cancer Society had already projected added pressures on cancer care services, predicting an increase of 79% in cancer cases by 2028–2032 [1]. COVID-19 has generated significant disruptions in oncology care provision that are likely to have lingering adverse effects across the cancer care trajectory for years to come. Canadian prediction models indicate that in the context of COVID-19, cancer care interruptions could account for an additional 21,247 (2.0%) cancer-related deaths in 2020–2030 [2]. Despite these precarious circumstances, continuation and optimization of cancer services remains an uncontested priority [3].

A systematic review has identified eight types of delays and/or disruptions in cancer care due to the COVID-19 pandemic: (1) treatment interruption, (2) treatment delay, (3) treatment change, (4) reduction in the number of treatments, (5) diagnostic interruption, (6) diagnosis delay, (7) reduction in the number of diagnoses, and (8) healthcare service disruption (due to personnel, supplies, settings, etc.) [4]. These challenges have amplified the demands on the already strained oncology workforce [5], which may further jeopardize future cancer care provision [6,7,8,9,10,11]. With the rising incidence of cancer and urgent need for physicians to deliver oncology treatments, general practitioners in oncology (GPOs) are poised to play a substantial role in mitigating the current crisis [12].

GPOs, also referred to as family practitioners in oncology, are specially trained family physicians with dedicated practices in oncology [13]. Numerous GPOs across Canada presently provide systemic regimen and radiation treatment supervision, management of physical and psychosocial treatment-related effects, as well as survivorship care [14]. BC Cancer and CancerCare Manitoba are recognized for their educational expertise and clinical integration of GPOs in their respective provinces. BC Cancer is renowned for its General Practitioner in Oncology Education Program, which was established in 2004 [15]. To date, nearly 100 family physicians from over 30 communities have completed the program and are actively working as GPOs in British Columbia [15]. Similarly, CancerCare Manitoba has pioneered GPO-led oncology services across the province, which efficiently provide cancer treatments to patients within the proximity of their homes [16]. These GPO-led services account for nearly 30% of all intravenous chemotherapy treatments annually [16].

The COVID-19 pandemic’s reverberating effects have led to a transformation in the landscape of oncology provision, which will undoubtedly continue to evolve. To support navigation through these changes, promoting an enhanced collaborative community amongst oncology providers, including GPOs, is imperative [17]. Moreover, providing high-quality evidence-based continuing education is also of utmost importance [18]. To that effect, the Canadian Association of General Practitioners in Oncology (CAGPO) is proud to partner with *Current Oncology* for its third educational series. The six-article series, produced by distinguished experts, will cover relevant and evidence-driven supportive care topics, as well as latest treatment advances in oncology.

CAGPO is a recognized national organization dedicated to supporting the educational needs of GPOs and allied health professionals. In addition to hosting an annual accredited conference, CAGPO provides a scholarship program for primary care providers who want to enhance their knowledge and clinical skills in the field of oncology. To learn more or to become of member of this community of practice, please visit: http://cagpo.ca.
